# Experiential Service Learning: Promoting Competency-based Education for Gerontology Students

**DOI:** 10.1093/geroni/igab046.2811

**Published:** 2021-12-17

**Authors:** Theresa Abah, Linda Paumer

**Affiliations:** California State University, Sacramento, Sacramento State University, California, United States

## Abstract

Service-learning is an effective pedagogical approach meant to deepen learners understanding of course content by linking scholarship and social action when structurally organized based on attainment of professional core competencies. The recent COVID-19 pandemic caused a rethinking of the way service learning practicum is delivered, especially as it relates to training health professionals who engage collaboratively with older persons (individuals aged 65 years and older). This poster examines the challenges faced by gerontology students as they transitioned to fully virtual learning and practicum format, the lessons learned, and how to close the gap between theory and practice for better programmatic processes. The data used to gather students experiences include; student reflective journals, class discussions and survey questions to students (n=44). The analytic framework utilized is the Social Cognitive Theory, (SCT)- which explains how individuals can master concepts through verbal and physical persuasion, including peer modelling. The goal is to promote leaners self- regulatory skills to achieve the course learning objectives, as obtained from the SCT six strategies for setting achievable goals, through: Feedback, self-instruction, self-monitoring, use of support and goal setting. Some of the lessons learned suggest students benefit more from service learning when they receive continuous feedback about how to develop intergenerational relationships with older adult partners assigned to in the community (68%), than from goal setting strategies (24%). The implication for practice is: there is a need to develop structured service-learning guidelines for undergraduate students in gerontology program to be prepared to better serve older adults.

